# Functional and morphological results of treatment of macula-on and macula-off rhegmatogenous retinal detachment with pars plana vitrectomy and sulfur hexafluoride gas tamponade

**DOI:** 10.1186/s12886-019-1120-3

**Published:** 2019-05-24

**Authors:** Dorota Borowicz, Katarzyna Nowomiejska, Dominika Nowakowska, Agnieszka Brzozowska, Mario D Toro, Teresio Avitabile, Anselm G. Jünemann, Robert Rejdak

**Affiliations:** 1 Department of General Ophthalmology, Medical University of Lublin, Poland, ul. Chmielna 1, 20-079 Lublin, Poland; 2 Department of Mathematics and Medical Biostatistics, Medical University, Lublin, Poland; 3 Department of Ophthalmology, University of Catania, Catania, Italy; 4 University Eye Hospital, Rostock, Germany; 5 Department of Experimental Pharmacology, Medical Research Centre, Polish Academy of Sciences, Warsaw, Poland

**Keywords:** Rhegmatogenous retinal detachment, Metamorphopsia, M-charts, Microperimetry

## Abstract

**Background:**

To examine morphological and functional results after pars plana vitrectomy (PPV) with sulfur hexafluoride (SF6) gas tamponade due to macula-on and macula-off rhegmatogenous retinal detachment (RRD) during 6 months of the follow-up.

**Methods:**

The study included 62 eyes that underwent successful PPV with SF6 tamponade with macula-on (34 eyes) and macula-off (28 eyes) RRD preoperatively. The best-corrected visual acuity (BCVA), Amsler test, M-charts, optical coherence tomography (OCT) and microperimetry were performed at 1, 3 and 6 months postoperatively.

**Results:**

Results of the Amsler test were abnormal postoperatively in 54% of the patients in the group with macula-off and in 32% of the patients with macula-on RRD. Horizontal M-charts improved significantly from 0.33 to 0.2, vertical M-charts– from 0.29 to 0.17 during 6 months of the follow-up. There was a significant increase in the central retinal thickness (CRT) and average thickness (AT) between follow-up examinations only in the macula-off group. 29 of 62 eyes (47%) after surgery (equally with macula-on and macula-off RRD) showed morphological changes in OCT in the macular region, as epiretinal membrane, macular edema, subretinal fluid or alterations of the outer layers of the retina. The average threshold in microperimetry increased significantly within both groups during the follow-up.

**Conclusion:**

Both horizontal and vertical M-charts scores, as were as microperimetry sensitivity improved significantly during the 6 months of the follow-up both in macula-on and macula-off group. Although PPV with SF6 gas tamponade was successful, almost half of eyes revealed anatomical changes in the macular region in OCT both with macula-on and macula-off group.

**Trial Registration:**

Current Controlled Trials NCT03902795 registered on 03/04/2019. Retrospectively registered.

## Background

Rhegmatogenous retinal detachment (RRD) is one of the most sight-threatening ophthalmological conditions caused by separation of neurosensory retina from the underlying retinal pigment epithelium (RPE) [1]. There is a high geographical variation of RRD with incidence ranging from 6.3 to 17.9 in 100.000 persons per year [2]. The pars plana vitrectomy (PPV) is one of the most effective procedures for the treatment of RRD with high anatomical success rate [3] - the mean postoperative reattachment rate being 93.3% [4]. Intraocular gas is commonly used as an adjunct during vitreoretinal surgery to provide the internal tamponade in the management of RRD [5, 6]. Although differentiation between macula-on and macula-off detachment is a standard clinical practice, there are no studies in medical literature which would compare detailed functional results of macula-on and macula-off retinal detachment.

Visual acuity is usually used in clinical practice as an outcome measure to evaluate the postoperative visual function but it does not always allow for a full assessment of visual function. Even after successful operation, the postoperative quality of vision, stereopsis and near vision may be unsatisfactory [7, 8]. Visual distortion and changes are also reported in optical coherence tomography (OCT) examination as a consequence of successful vitreoretinal surgery due to RRD [9].

The Amsler grid has been used so far for the qualitative assessment of the metamorphopsia since 1947 [10]. Matsumoto developed M-charts for quantitative metamorphopsia evaluation in macula diseases in 1990 [11]. Microperimetry is the method for assessing the retinal sensitivity imposed on the eye fundus. Both methods have been used to assess progress of the disease and effectiveness of therapy in retinopathies and maculopathies of different origin [12, 13].

The purpose of this study was to analyze functional and structural results during 6 months of the observation after successful repair of RRD with PPV and SF6 gas tamponade.

## Methods

Sixty-two patients with RRD were included in this prospective study from March 2016 to October 2017 in the Department of General Ophthalmology in Lublin, Poland. The study was performed in accordance with the Declaration of Helsinki. Ethics Committee at the Medical University of Lublin approved this study (number of approval KE-0254/12/2017). All participants provided their written informed consent to the study. Exclusion criteria were as follows: history of uveitis, diabetic retinopathy, previous vitreoretinal surgery, glaucoma or eye trauma, also RRD combined with macular hole or proliferative vitreoretinopathy. The study included 25 females and 37 males, their mean age was 62.27 ± 10.96 years. The patients were divided into two groups: the first group consisted of 34 eyes (55%) with macula-off RRD and the second group consisted of 28 eyes (45%) with macula-on RRD. macula-on RRD was defined as a condition where the fovea is not involved at presentation.

PPV was performed by two vitreoretinal surgeons (RR and KN) under peribulbar anaesthesia using Constellation system (Alcon, Fort Worth, Texas, US). The surgical technique included 23 gauge (38 eyes) or 25 gauge (22 eyes) complete PPV with perfluorodecaline, drainage of the subretinal fluid, fluid-air exchange, endolaser around retinal tears and gas tamponade with 25% sulfur hexafluoride (SF6) gas. No internal limiting membrane peeling was done during PPV. In 49 eyes PPV was combined with phacoemulsification and acrylic foldable intraocular lens (IOL) implantation with 2.2 mm corneal incision.

All patients underwent BCVA measurements (logMAR charts), slit lamp examination, applanation tonometry, Amsler test, vertical and horizontal M-charts test, microperimetry (MAIA CenterVue, Italy), dilated fundoscopy and OCT-2000 examination (Topcon Corporation, Tokyo, Japan). All patients have were examined 1, 3 and 6 months after the surgery.

Central retinal thickness (CRT) measurements were performed with three-dimensional (3D) OCT-2000 (Topcon Corporation, Tokyo, Japan). Each eye was examined after pupillary dilatation. Macular 3D scan protocol (6 mm × 6 mm area centered on the fovea with a scan density of 512 [vertical] × 128 [horizontal] scans) was used in all the patients. The retinal images were collected and assessed as normal, epiretinal membrane, subretinal fluid or IS/OS disruption.

To assess metamorphopsia, the standard Amsler test was performed with black lines on the white background. Additionally, M-charts were used. The M-charts consist of 19 dashed lines with increasing distance between points in the range of 0.2 ° to 2.0 ° viewing angle. In the center of each line, there was a fixation point of 0.3°. The examiner presented consecutive dotted lines, starting with a solid line (0°) and the patient had to state whether the presented line is distorted or not. The patients with macular disorders perceive the straight line as irregular at first; as the distance between the dots increases, the irregularity of the line decreased until the line became straight. This is recorded as a metamorphopsia score (from 0.2 to 2.0). The test was carried out both horizontally and vertically after 180° rotation with proper near correction.

The retinal sensitivity and fixation indexes were assessed in microperimetry. The background luminance was 4 asb, stimulus intensity range was 0–36 dB, Goldmann III stimulus size, stimulus duration was 200 ms and testing protocol was 4–2 threshold strategy.

Statistical analysis was performed using STATISTICA 13.0 software (StatSoft, Krakow, Poland). All values are presented as the median ± standard deviation. Non-parametric tests have been used for statistical analysis, as the number of patients in the groups was small (*n* = 34 and 28) and the conditions for parametric tests were not fulfilled (normality of distribution, homogenity of the variance). The Shapiro-Wilk test analyzed measurable parameters. The Mann-Whitney U test was used to compare two independent groups. The Friedman ANOVA test was used to compare the results from 1 month to 6 months and before surgery. *P* < 0.05 was considered to be of statistical significance.

## Results

### Visual acuity

Before the surgery the median BCVA (logMAR) was 1.20 ± 0.69 overall. The median preoperative BCVA (logMAR) in a group with macula-off RRD was 1.70 ± 0.18 and in the group patients with macula-on it was 0.30 ± 0.34 before the surgery. Thus, the visual acuity was significantly worse in a group with macula-off RRD (*p* < 0,000001).

**Fig. 1:**
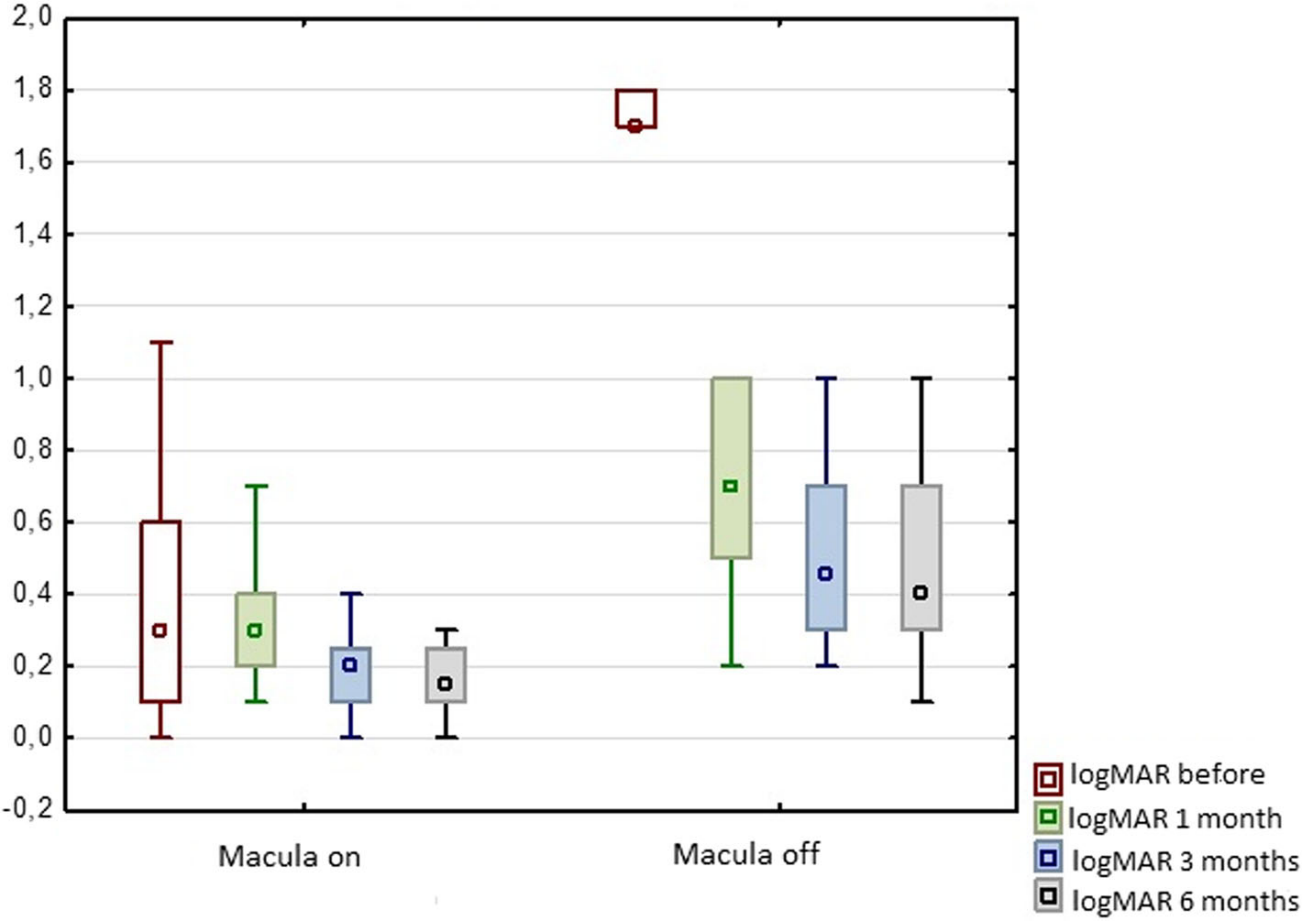
Median best corrected visual acuity (BCVA) in logMAR scale before and 1, 3 and 6 months after the vitrectomy due to rhegmatogenous retinal detachment in two groups of patients with macula-on and macula-off preoperatively

The median final postoperative BCVA (logMAR) in all patients was 0.30 ± 0.29; in the group with macula-off it was 0.40 ± 0.28 and in the group with macula-on it was 0.15 ± 0.20. Postoperative visual acuity improved significantly during the 6 months after the surgery in both groups. Statistical analysis showed a significant improvement in visual acuity after the surgery in the group with the macula- off (*p* < 0.000001) and in the group with the macula-on (*p* < 0.000001). There were also significant differences in BCVA before surgery (p < 0.000001), 1 month after the surgery (*p* = 0.000002), 3 months after the surgery (*p* = 0.000001) and 6 months after the surgery (*p* = 0.000004) (Fig. 1).

### Optical coherence tomography

**Fig. 2:**
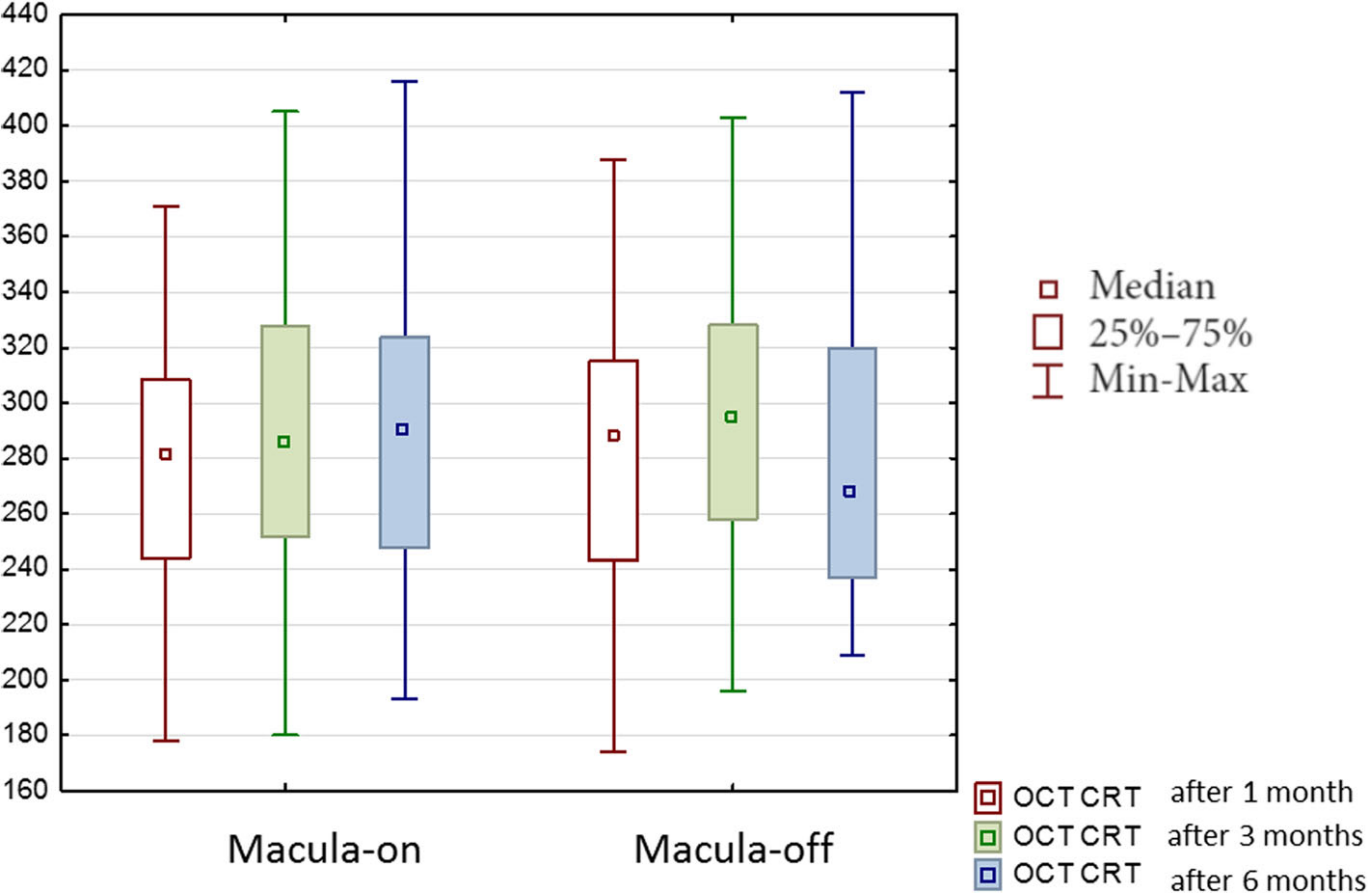
Median central retinal thickness (CRT) in micrometers obtained with optical coherence tomography during 6 months of the follow-up after vitrectomy due to rhegmatogenous retinal detachment

There were significant differences in CRT among follow-up examinations (*p* = 0.02). The median was 285.0 ± 47,91 μm after 1 month, 289,50 ± 63.26 μm after 3 months and 279,00 ± 56.67 μm after 6 months. In the group with the macula-on there were no significant changes during the follow-up (*p* = 0.39), but in the group with macula-off the changes were significant (*p* = 0.009) (Fig. 2).

**Fig. 3:**
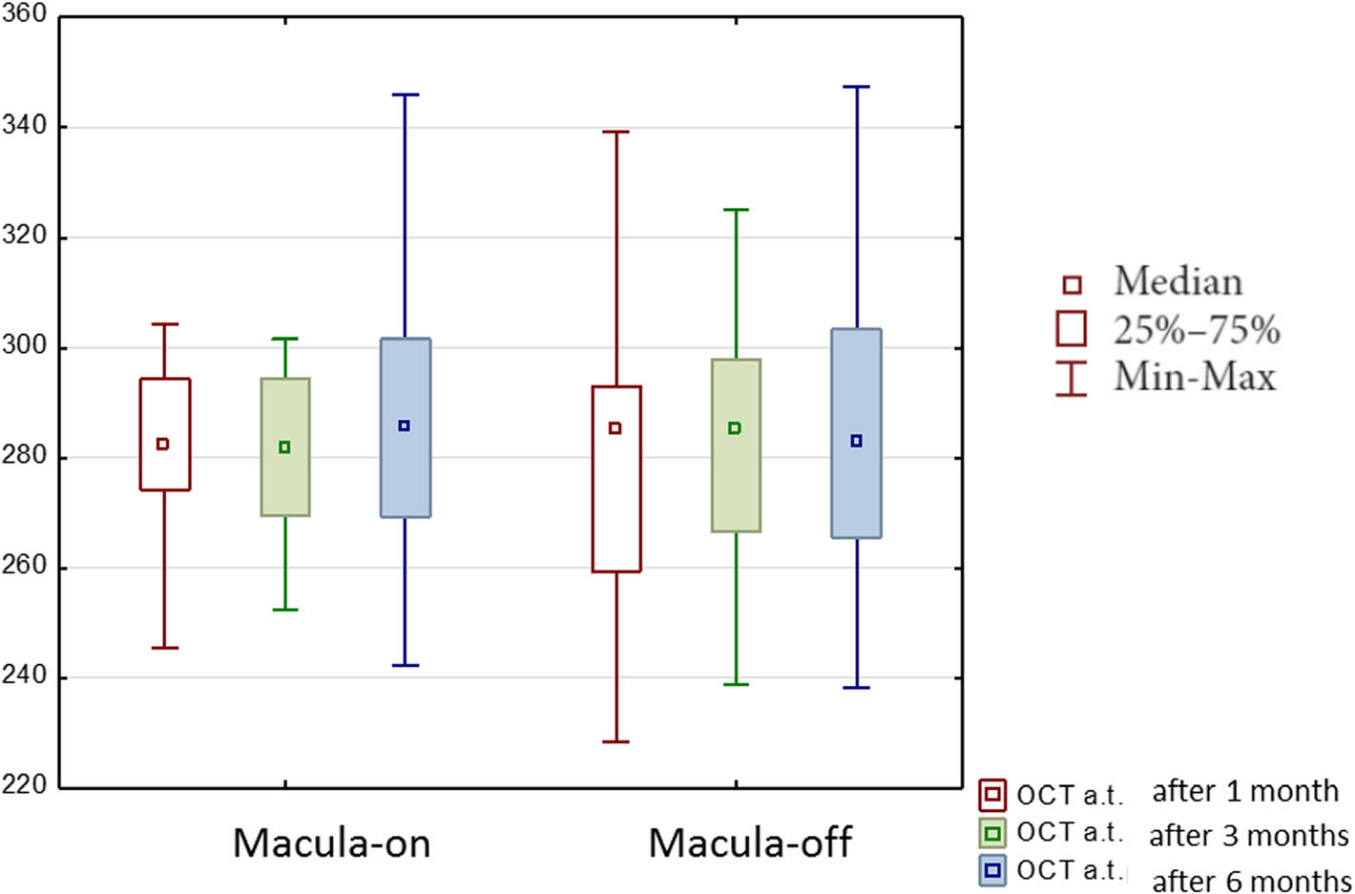
Median average thickness (AT) in micrometers obtained with ocular coherence tomography during 6 months of the follow-up after vitrectomy due to rhegmatogenous retinal detachment

The median AT of all groups was 284.85 μm ± 21.72 μm after 1 month, 284.90 μm ± 28.74 μm after 3 months and 284.70 μm ± 26.70 μm after 6 months. Statistical analysis showed significant differences in AT in the respective periods after PPV in the group with macula-off (*p* = 0.03), while in the group of patients with macula-on no significant differences were found (Fig. 3).

In 29 out of 62 eyes morphological changes of the macular region were found in OCT, such as a epiretinal membrane (14 eyes), macular edema (15 eyes), subretinal fluid (2 eyes), IS/OS disruption (3 eyes) were observed (more than one possible) both in macula-off (14 eyes) and macula-on RRD (15 eyes) (Fig. 3).

### Metamorphopsia assessment

The results of the Amsler test were abnormal in 32% of the patients with macula-on and in 54% of the patients with macula-off group. Statistical analysis did not show any significant differences between the Amsler test results in the follow-up period (*p* > 0.05). During the final examination after 6 months most of the Amsler test results were negative (61%).

**Fig. 4:**
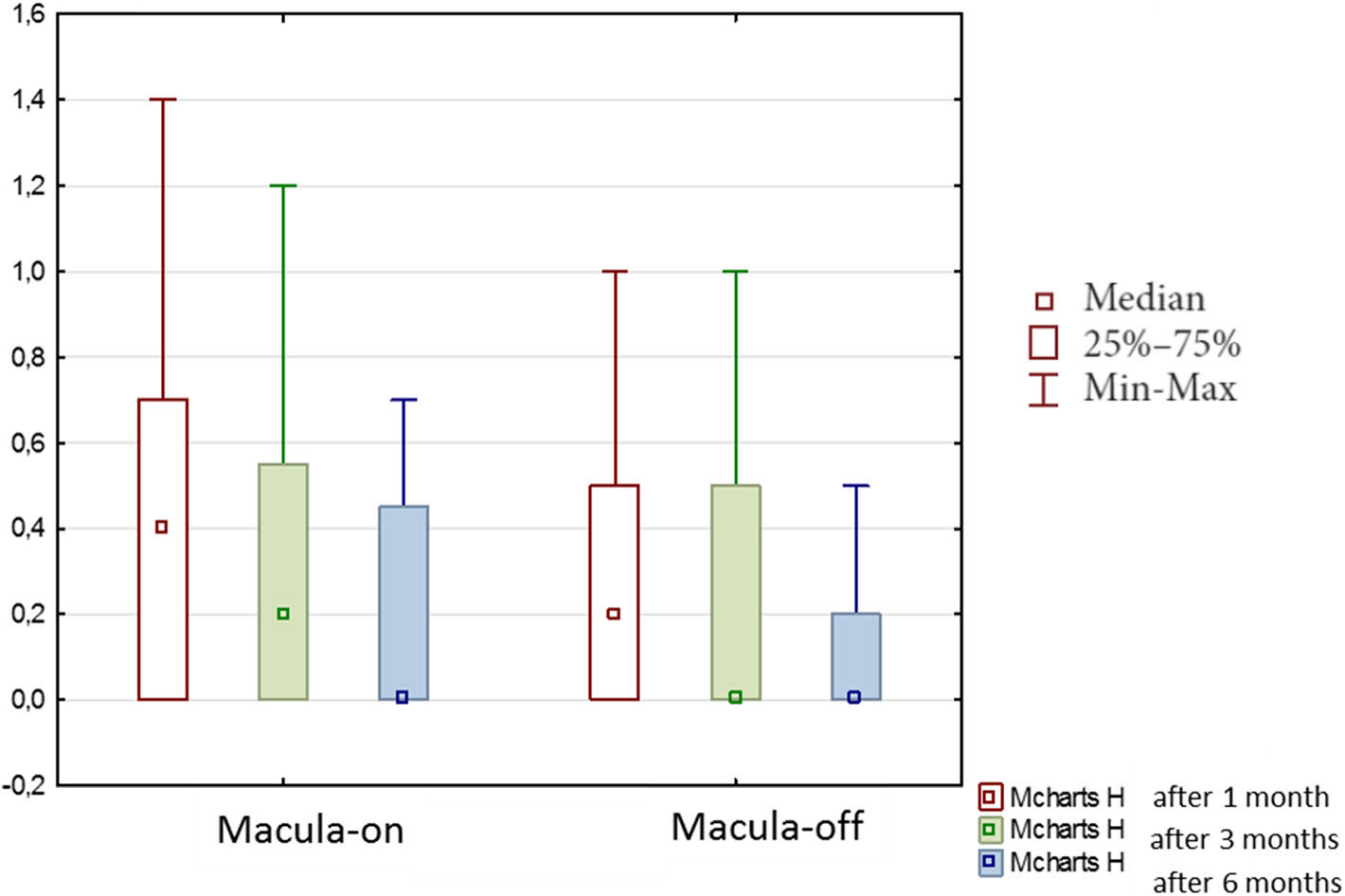
Median horizontal metamorphopsia (M-charts H) scores during 6 months of the follow-up after vitrectomy due to rhegmatogenous retinal detachment

Finally, M-charts results were abnormal in 60% of the patients with macula-on group and in 62% of the patients with macula-off RRD. The median horizontal metamorphopsia score of all patients was 0.20 ± 0.38 after 1 month, 0.2 ± 0.32 after 3 months, 0.00 ± 0.28 after 6 months, while horizontal metamorphopsia in patients with macula-on was 0.40 ± 0.36 after 1 month, 0.20 ± 0.29 after 3 months, 0.00 ± 0.24 after 6 months, these differences were statistically significant (*p* < 0.00001). Horizontal metamorphosia in group with macula-off was 0.20 ± 0.41 after 1 month, 0.00 ± 0.34 after 3 months, 0.00 ± 0.31 after 6 months (Fig. 4). Statistical analysis showed significant differences in the assessment of horizontal M-charts after the surgery in both groups (*p* < 0.000001).

**Fig. 5:**
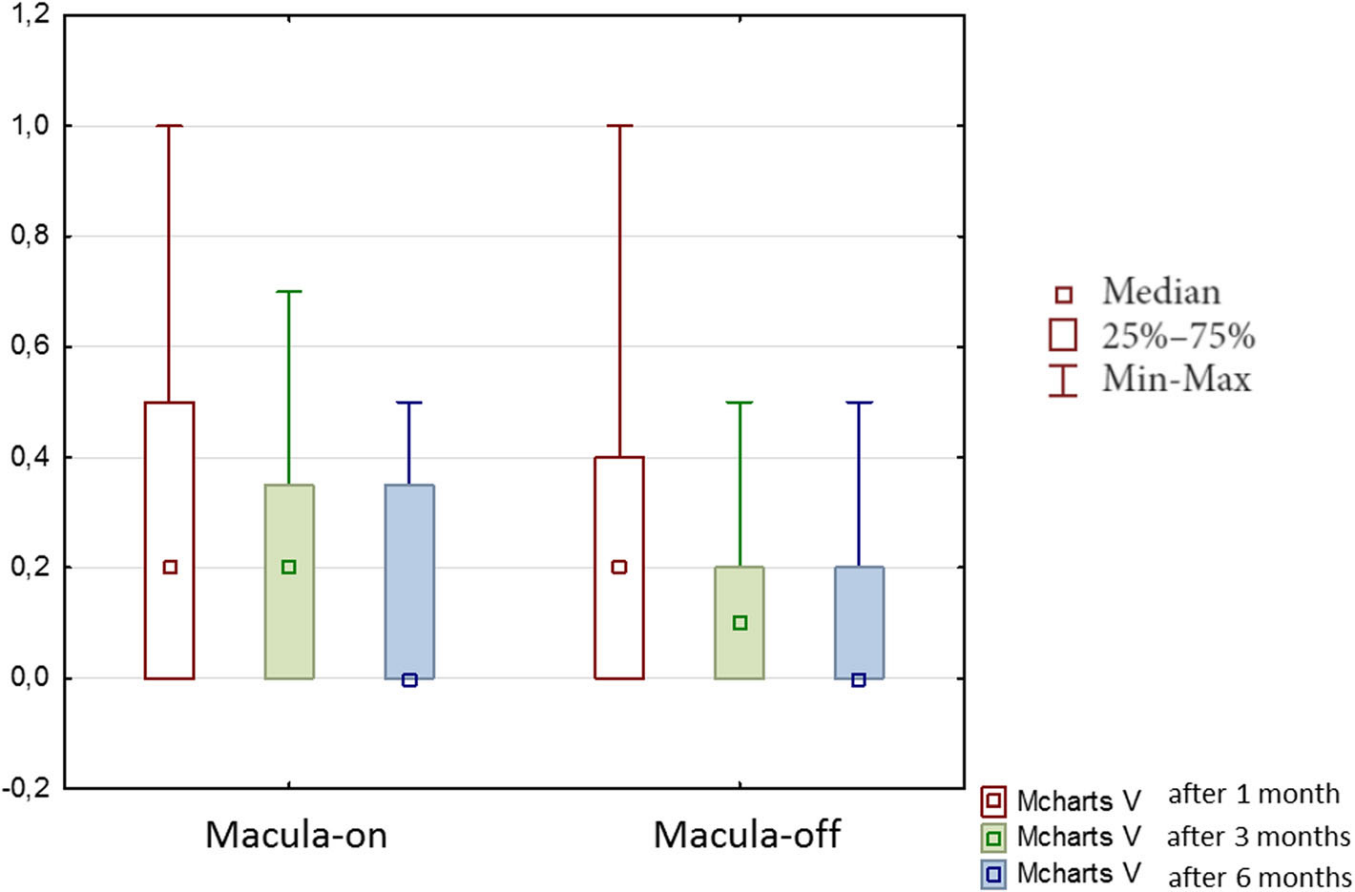
Median vertical metamorphopsia (M-charts V) scores during 6 months of the follow-up after vitrectomy due to rhegmatogenous retinal detachment

Generally, the median vertical metamorphopsia score of all patients was 0.20 ± 0.32 after 1 month, 0.20 ± 0.27 after 3 months, 0.00 ± 0.24 after 6 months, these differences were statistically significant (*p* < 0.00001). The median vertical metamorphopsia scores of patients with macula-on was 0.20 ± 0.35 on 1 month, 0.20 ± 0.29 on 3 months, 0.00 ± 0.24 on 6 months and vertical metamorphopsia of patients with “macula-off” was 0.2 ± 0.30 on 1 month, 0.10 ± 0.25 on 3 months, 0.00 ± 0.25 on 6 months (Fig. 5). Statistical analysis showed significant differences in the assessment of vertical M-charts after the surgery in the group with the macula-off (*p* < 0.000001) and in the group with the macula-on (*p* < 0.000001).

### Microperimetry

**Fig. 6:**
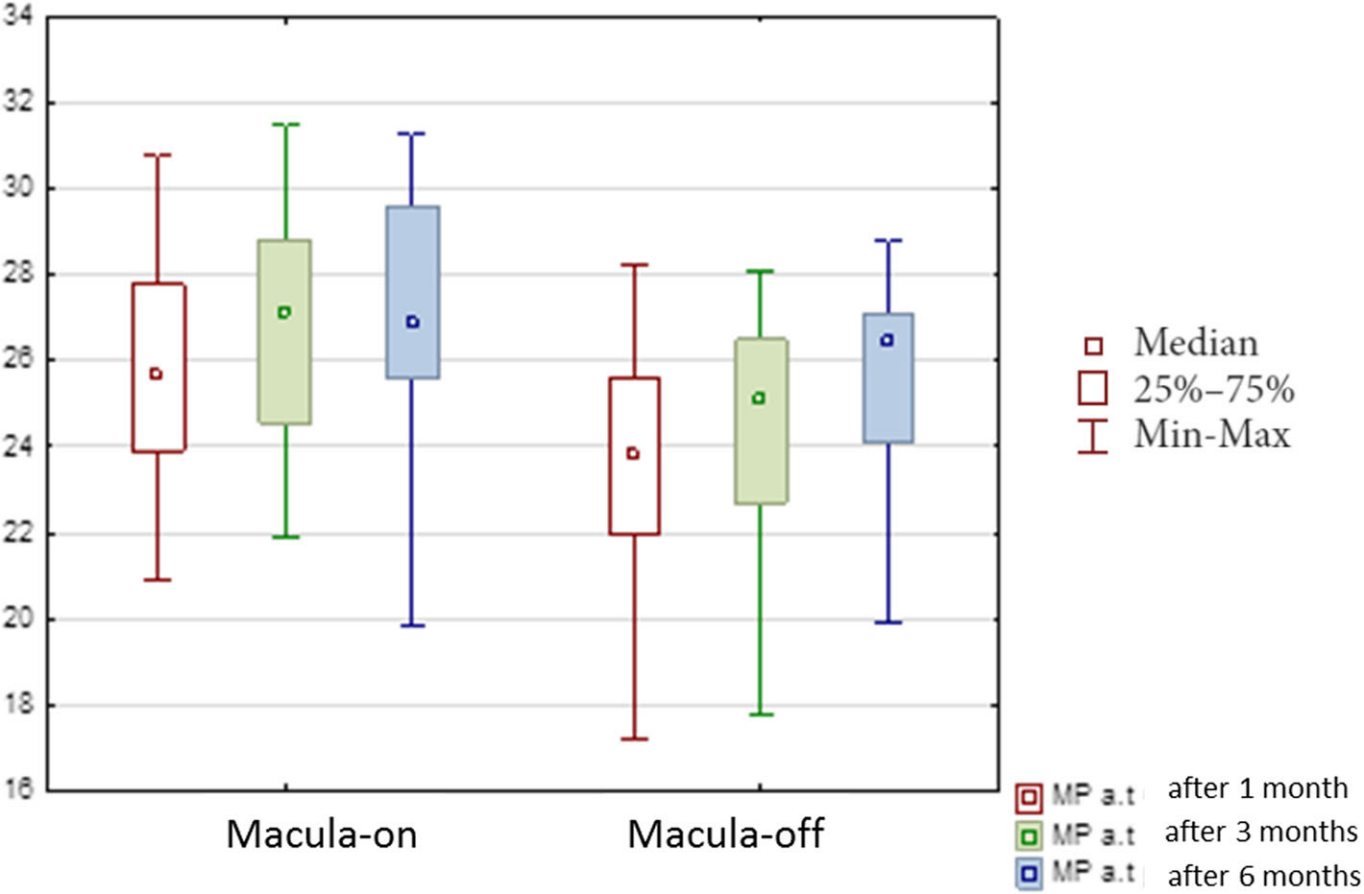
Median average threshold (AT) in decibels of retinal sensitivity in microperimetry during 6 months of the follow-up after vitrectomy due to rhegmatogenous retinal detachment

The median of retinal sensitivity in all patients was 24.90 ± 4.33 dB after 1 month, 25.90 ± 3.51 dB after 3 months and 26.55 ± 3.89 dB after 6 months. The median of retinal sensitivity in the group with macula-on was 25.65 ± 3.90 after 1 month, 27.15 ± 3.14 dB after 3 months, 26.85 ± 3.40 dB after 6 months while the median of retinal sensitivity in macula-off group was 23.80 ± 4.41 dB after 1 month 25.10 ± 3.53 dB, after 3 months and 26.10 ± 4.18 dB after 6 months (Fig. 6).

Statistical analysis showed significant differences in retinal sensitivity after the surgery in the group with the macula-off (*p* < 0.000001) and in the group with the macula-on (*p* < 0.001). There were also significant differences between the two groups in the assessment of retinal sensitivity 1 month (*p* = 0.017) and 3 months (*p* = 0.003) after the PPV while 6 months after the surgery the differences were not significant (*p* = 0.09).

Median fixation stability with P1 and P2 values in microperimetry for all patients was P1 = 0.88 ± 0.27 and P2 0.97 ± 0.16 after 1 month, P1 = 0.94 ± 0.22 and P2 = 0.99 ± 0.10 after 3 months and P1 = 0.91 ± 0.18 and P2 = 0.99 ± 0.11 after 6 months. These differences were to some extent statistically significant for P1 values (*p* = 0.02).

## Discussion

In the present study the functional and morphological outcomes with M-charts and microperimetry were assessed in the eyes of patients after PPV with SF6 gas due to RRD with macula-on and macula-off during 6 months of the follow-up period.

There are only few studies comparing the results of surgical treatment of macula-on and macula-off RRD, they are summarized in Table 1. Rezar and colleagues [17] described results of 23-gauge vitrectomy in pseudophakic RRD, 1, 3, 6 and 12 months after the surgery. They concluded that macula-off RRDs showed delayed visual rehabilitation and macula-on RRDs showed significantly more improvement than macula-off RRDs. In the latest study Potic and co-workers analyzed risk factors for macular involvement in RRD [18]. They found that pseudophakic lens status and axial length < 25 mm were independent predictive factors for macula-off RD. They described a total of 52.6% (102/194) patients with macula-off RRD. It is almost the same proportion as in our study.

**Table 1:** Summary of papers describing metamorphopsia after surgery due to rhegmatogenous retinal detachment (RRD)

Authors of publication, year	Number of eyes	Rhematogenous retinal detachment		Type of surgery	Observation time (months)	Metamorphopsia assessment			Abnormalities in optical coherence tomography
		macula-on	macula-off			Method	macula-on	macula-off	
Wang et al., 2005 [14]	46	not measured	46	scleral buckling burgery	2	Amsler grid	not measured	67%	64%
Okamoto et al., 2014 [15]	129	69	60	scleral buckling or vitrectomy	12	M-charts	13%	68%	39%
Murakami et al., 2018 [16]	47	17	30	pars plana vitrectomy	12	M-charts	35%	70%	72%
Okuda et al., 2018 [9]	40	14	26	pars plana vitrectomy	12	M-charts	21%	64%	not measured
Present study	62	28	34	pars plana vitrectomy	6	M-charts	60%	62%	47%

Ross and co-workers showed three important factors for visual recovery after PPV: good pre-operative BCVA, short duration of RRD and younger age [19]. Liu and colleagues observed that preoperative visual acuity of more than 0.1 and patients age under 60 had prognostic value on the final visual activity after treatment RRD [20]. Our patients’ mean age was 62.27 ± 10.96 and mean BCVA (logMAR) improved from preoperative 1.10 ± 0.69 to 0.51 ± 0.30 after 1 month and 0.36 ± 0.29 after 6 months.

Metamorphopsia is a one of the most common postoperative symptoms after vitreoretinal surgery due RRD. Measuring metamorphopsia may be useful in macular diseases such as: macular hole, epiretinal membrane [21], age-related macular degeneration [22], branch retinal vein occlusion [23], central retinal vein occlusion [24] and central serous chorioretinopathy [25]. The Amsler test can be used to assess the quality metamorphopsia but the innovative method M-charts gives a quantitative assessment. In our study the results of the Amsler test were abnormal postoperatively in 54% of patients in a group with macula-off and in 32% of patients with macula-on RRD. M-charts results were incorrect in 62% of patients with macula-off RRD and in 60% of patients in macula-on group.

The similar to our results in regard to metamorphopsia scores have been obtained by studies of Wang and co-workers [14], Okamoto and co-authors [15], Murakami and colleagues [16] and Okuda et al. [9] (Table 1).

Arimura and co-workers reported that using M-charts more vertical retinal contraction correlated with increasing horizontal metamorphopsia and more horizontal retinal contraction correlated with increasing vertical metamorphopsia [26].

OCT is a useful noninvasive diagnostic method, which provides information about morphological changes in macula after surgical interventions. Wakabayashi and co-authors examined 53 eyes with macula-on (15 eyes) and macula-off RRD (38 eyes) after successful surgical repair. They observed foveal anatomic abnormalities in 62% of the patients, such as disruption of the junction between the photoreceptor inner and outer segments (43%), epiretinal membranes (12%), subretinal fluid (11%), macular edema (4%). Disruption of the photoreceptor IS/OS junction was observed only in macula-off eyes [27]. In our study 29 out of 62 eyes (47%) after surgery showed morphologic changes in the macular region in OCT, such as a epiretinal membrane, macular edema, subretinal fluid, which were reduced during the next examination. Benson and colleagues observed subretinal fluid in 15% of patients after PPV with gas tamponade due RRD; it was still present in 53% during 6-months follow-up period [28]. Cheng and co-workers observed increased central macular thicknesses which may have been due to the subretinal fluid in patient after surgical interventions (PPV, SB) due to RRD. They observed faster absorption of SRF after PPV than after SB [29]. In our study CRT increased significantly after 6 months. Okamoto and colleagues observed morphological changes in OCT after PPV (98 patients) and scleral buckling (31 patients) in 18 patients with metamorphopsia after surgery and in 9 patients with macula-on RRD.

Microperimetry can provide useful information about the visual function in patients after PPV due to RRD. Rossetti and colleagues analyzed 6 eyes after successful buckling surgery for macula-off RRD. They observed retinal sensitivity in 121 microperimetry (MP-1) after 1, 3, 6, 12, and 18 months. The MS in normal subjects can range from 16 to 20 (mean 19.7 ± 0.8 dB); using the MP-1 Macular sensitivity (MS) increased from 9.7 ± 7.1 after 1 month to 13.5 ± 5.6 dB at the final check and was weakly correlated with visual activity and OCT. Fixation was stable in 5 eyes. Marked decrease in MS and IS/OS junction photoreceptor disruption was found only in one eye with SRF [30]. In our study retinal sensitivity increased from 23.77 ± 4.33 dB to 26.07 ± 3.89 dB after 6 months after PPV. Lai and co-authors noticed that the areas of decreased sensitivity in microperimetry corresponded to changes in OCT and abnormal fundus autofluorescence imaging [31].

## Conclusions

Summing up, both horizontal and vertical M-charts scores, as were as sensitivity and fixation pattern in microperimetry improved significantly during 6 months of the follow-up both in macula-on and macula-off RRD. There was a high percentage of eyes with macular changes in OCT examination after PPV both with macula-off and macula-on eyes.

## Abbreviations

AT: average thickness
BCVA: best-corrected visual acuity
CRT: central retinal thickness
IOL: intraocular lens
MP: microperimetry
MS: Macular sensitivity
OCT: optical coherence tomography
PPV: pars plana vitrectomy
RPE: retinal pigment epithelium
RRD: rhegmatogenous retinal detachment
SB: scleral buckling
SF6: sulfur hexafluoride
SFR: subretinal fluid
